# Mapping Salivary Proteases in Sjögren’s Syndrome Patients Reveals Overexpression of Dipeptidyl Peptidase-4/CD26

**DOI:** 10.3389/fimmu.2021.686480

**Published:** 2021-06-17

**Authors:** Laís Garreto, Sébastien Charneau, Samuel Coelho Mandacaru, Otávio T. Nóbrega, Flávia N. Motta, Carla N. de Araújo, Audrey C. Tonet, Flávia M. B. Modesto, Lilian M. Paula, Marcelo Valle de Sousa, Jaime M. Santana, Ana Carolina Acevedo, Izabela M. D. Bastos

**Affiliations:** ^1^ Pathogen–Host Interface Laboratory, Department of Cell Biology, Institute of Biology, University of Brasília, Brasília, Brazil; ^2^ Laboratory of Protein Chemistry and Biochemistry, Department of Cell Biology, Institute of Biology, University of Brasília, Brasília, Brazil; ^3^ Faculty of Ceilândia, University of Brasília, Brasília, Brazil; ^4^ Laboratory of Immune Gerontology, Catholic University of Brasília, Brasília, Brazil; ^5^ Department of Dentistry, Paulista University, Brasília, Brazil; ^6^ Laboratory of Oral Histopathology, Department of Odontology, Health Sciences Faculty, University of Brasília, Brasília, Brazil

**Keywords:** saliva, Sjögren’s syndrome, DPP4/CD26, protease inhibitors, protease activity, proteome

## Abstract

Sjögren’s Syndrome (SS) is an autoimmune exocrinopathy characterized by the progressive damage of salivary and lacrimal glands associated with lymphocytic infiltration. Identifying new non-invasive biomarkers for SS diagnosis remains a challenge, and alterations in saliva composition reported in patients turn this fluid into a source of potential biomarkers. Among these, proteases are promising candidates since they are involved in several key physio-pathological processes. This study evaluated differentially expressed proteases in SS individuals’ saliva using synthetic fluorogenic substrates, zymography, ELISA, and proteomic approaches. Here we reported, for the first time, increased activity of the serine protease dipeptidyl peptidase-4/CD26 (DPP4/CD26) in pSS saliva, the expression level of which was corroborated by ELISA assay. Gelatin zymograms showed that metalloproteinase proteolytic band profiles differed significantly in intensity between control and SS groups. Focusing on matrix metalloproteinase-9 (MMP9) expression, an increased tendency in pSS saliva (p = 0.0527) was observed compared to the control group. Samples of control, pSS, and sSS were analyzed by mass spectrometry to reveal a general panorama of proteases in saliva. Forty-eight protein groups of proteases were identified, among which were the serine proteases cathepsin G (CTSG), neutrophil elastase (ELANE), myeloblastin (PRTN3), MMP9 and several protease inhibitors. This work paves the way for proteases to be explored in the future as biomarkers, emphasizing DPP4 by its association in several autoimmune and inflammatory diseases. Besides its proteolytic role, DPP4/CD26 acts as a cell surface receptor, signal transduction mediator, adhesion and costimulatory protein involved in T lymphocytes activation.

## Introduction

Sjögren’s Syndrome (SS) is a systemic autoimmune disease characterized by the destruction of exocrine glands, mainly salivary and lacrimal. With a prevalence of approximately 0.5% in the general population, SS is one of the most frequent autoimmune disorders in women (nine in 10 patients are women). It can be defined as primary SS (pSS) or associated/secondary SS (sSS) if combined with another systemic autoimmune disease (1, 2). SS’s main symptom is dryness of oral mucosa and eyes due to decreased secretion of saliva and tears (3). Oral manifestations include hyposalivation and an increased risk for cervical dental caries and dental erosion (4), oral candidiasis, angular cheilitis, and severe swallowing and speech difficulties (5). Moreover, extra-glandular manifestations can occur in the cardiovascular, respiratory, and digestive systems (6). Disease severity associated with B cell lymphoma development, organ-specific manifestations, and infections can lead to excess mortality (7).

SS glandular lesions are due to massive infiltration of inflammatory cells (including T and B lymphocytes, natural killer cells, dendritic cells, and macrophages) and the formation of ectopic germinal centers (8–11). So far, SS etiology involves poorly elucidated genetic factors. Human leukocyte antigen (HLA) class II genes, mainly HLA-DRB and HLA-DQA alleles, seem to produce genetic susceptibility to SS. They may favor autoantibody synthesis against the Ro/La system (anti-Ro/SSA and anti-La/SSB) (12). These autoantibodies recognize different epitopes on the Ro/La proteins associated with small RNAs situated in the cytoplasmic and nuclear compartments (13). Also, in SS, miRNA differential expression has been reported (14). Recently, miR200b-5p expression in the minor salivary glands was related to the prediction of lymphomagenesis in SS patients (15). The SS etiology may also encompass environmental factors such as viral infections (16). The Human T Lymphotropic Virus Type 1 (HTLV-1) may trigger the disease, considering that antibodies for HTLV-1 were highly identified from pSS individuals’ sera in endemic areas (17, 18).

SS diagnosis relies on subjective and objective criteria (19, 20). These include clinical information collected from the patient’s history, autoantibodies in the serum, especially anti-Ro/SSA and anti-La/SSB, imaging analysis, and histopathological analyses of minor salivary glands. Occasionally, there is no autoantibody detection, mainly in the disease’s early stage (21). Also, the autoantibodies might be identified in mothers who gave birth to infants with neonatal lupus (22). Moreover, the autoantibodies are not exclusive to SS pathology. For instance they are also present in the serum of systemic lupus erythematosus patients (23). Thus, despite autoantibody detection, a biopsy of minor salivary glands remains necessary (13).

Studies comparing salivary protein composition between healthy individuals and patients of pathological conditions have identified potential disease biomarkers (24–28). Additionally, a diagnosis based on salivary samples is a non-invasive, low-cost method that can reduce anxiety and discomfort to the patient when submitted to biopsy. It also allows for more effective longitudinal monitoring (29). Either for oral or systemic disorders, such as periodontitis, cancer, AIDS, and even SS, saliva has been considered a potential tool for diagnosis (30–33).

Among potential saliva-derived biological markers, proteases are promising candidates since they are involved in several fundamental physiological processes, and their activity is tightly controlled through numerous redundant mechanisms (34). Proteolysis regulates vital mechanisms, such as embryological development, immune response, blood clotting and whole-body metabolism (35). Also, studies have related protease activities with autoimmune diseases. For instance metalloproteinase-9 (MMP9) is augmented in the sera of systemic lupus erythematosus patients (36) and dipeptidyl peptidase-4/CD26 (DPP4/CD26) in sera of type 1 diabetes, rheumatoid arthritis, systemic lupus erythematosus and inflammatory bowel disease (37).

To date, few studies have associated SS and salivary proteases (38, 39). The purpose of the present study was to analyze the proteolytic profile in stimulated whole saliva (SWS) protein samples from healthy individuals and patients with pSS or sSS using synthetic fluorogenic substrates, zymography, ELISA and mass spectrometry (MS). Here we propose that the protease DPP4/CD26 could be further explored as a potential biomarker in the saliva of SS patient diagnosis. Moreover, our proteomic analysis resulted in 208 identified protein groups. Among them, the serine proteases neutrophil elastase (ELANE), cathepsin G (CTSG) and myeloblastin (PRTN3) were found only in the saliva from SS patients.

## Materials and Methods

The present study was conducted following the principles of the Declaration of Helsinki. The Research Ethics Committee of the Faculty of Health Sciences, University of Brasilia, approved all procedures (CEP/FS, 073/11). All individuals provided written informed consent before participating in the study.

### Study Design

A control–case study was performed with 40 adults matched by age, gender, and socioeconomic situation. The control group was composed of 20 healthy individuals. They had no history of dry eyes or mouth, no salivary gland disease, and they were not taking any drugs associated with xerostomia, such as anti-depressives, birth control pills, and hormonal replacement. The affected group was composed of 20 SS individuals, 10 primary-SS (pSS) and 10 secondary-SS (sSS). They were diagnosed following with the American-European Consensus (19). The study was carried out with 38 women, one man in the SS group and one man in the control group. Exclusion criteria were all patients diagnosed with HIV, HCV, HVB, or HTLV-1 infections, diabetes, sarcoidosis, lymphoma, smokers, patients with a history of head and neck radiotherapy treatment, and patients using anticholinergic drugs. The manifestation of other systemic autoimmune diseases was registered. Clinical information about the appearance of oral mucosa (normal or dry), the clinical appearance of tongue (normal, dry, atrophic, atrophic with fissures or savory), the saliva aspect (normal, sticky or foamy) and the clinical presence of candidiasis (no signs, angular cheilitis or erythematous lesions) were collected (**Supplementary Table 1**).

### Saliva Collection and Sample Preparation

Patient samples of stimulated whole saliva (SWS) were obtained after a 2-h fast between 9 am and 11 am to reduce the influence of circadian rhythm, followed by chewing 1 g of flavorless chewing gum for 5 min (3). They did not receive any diet recommendations prior to fasting start. Patients were instructed to do not drink water and to do not perform oral hygiene until 2 h before saliva collection. During the SWS collection, they all received instructions to chew the unflavored chewing gum in both sides. Every 30 s, they let the saliva drain to the tube until the end of the 5 min. Salivary flow rate was also registered. During collection, the samples were kept on ice to prevent proteolysis. SWS samples were centrifuged at 4°C (2,600 × g, for 15 min) and stored at −80°C. In all proteolytic assays, SWS samples were used without any chemical processing or dilution.

### Protein Concentration

Total salivary protein measurement was performed by fluorometric assay (Invitrogen Qubit® 2.0 Fluorometer) with the Invitrogen™ Qubit™ Protein Assay Kit.

### Enzymatic Assays Using Fluorogenic Substrates

The proteolytic activity was assessed in saliva samples through fluorogenic substrates. Twelve substrates were tested in one individual from the control group, in triplicate: Gly-Pro-AMC, Phe-Arg-AMC, N-Gly-Gly-Arg-AMC, Pro-AMC, Arg-Arg-AMC, Ala-Ala-Phe-Ala-AMC, N-Suc-Ile-Leu-Cys-Ala-AMC, N-Suc-Gly-Pro-Leu-Gly-Pro-AMC, Gly-Arg-AMC, N-Suc-Leu-Thr-AMC, Arg-AMC, N-Suc-Leu-Leu-Val-Tyr-AMC. The enzymatic activity from saliva was measured by the releasing of 7-amino-4-methylcoumarin (AMC) from substrates (40) in a 96-well microplate in SpectraMax® M5 ROM v3.0.22 Molecular Devices spectrofluorometer at 380 nm excitation and 460 nm emission for 15 min. The rate of fluorescence liberated was calculated per minute (FU/min). All enzymatic reactions were performed in activity buffer (HEPES 25 mM, pH 7.5) in the presence of 20 µM of the substrate. The substrate Gly-Pro-AMC was tested in all saliva samples.

For proteolytic specific inhibition, protease inhibitors (**Supplementary Table 2**) were tested in an individual from the control group, by incubation for 15 min before adding the substrate.

### Gelatinase Activity

Gelatinase activity was analyzed by zymography. SWS (10 µl) was loaded on 8% SDS-PAGE gel copolymerized with 0.1% (w/v) gelatin. Gels were submitted to two washes within Tris-HCl 100 mM pH 7.5 and Triton 2.5% (v/v) for 30 min per wash followed by one wash with deionized water to remove Triton X-100. Next, they were incubated under 37°C overnight in Tris-HCl 100 mM pH 7.5 and stained within a fixing solution of Coomassie Blue R-250, for 1 h at room temperature (41). Samples were also subjected to a 10% SDS-PAGE to visualize the electrophoretic profile, mirrored to every zymogram. We tested the specific inhibitors 1 mM AEBSF, 100 µM E-64, and 1 mM EDTA for specific proteolytic gelatinase inhibition. Since EDTA is a reversible inhibitor, the gel strip treated with EDTA had its final concentration maintained in washing and incubation at 37°C overnight (**Supplementary Table 3**). The activity was determined by densitometry, using ImageJ 1.51a software for each gelatinolytic band’s mean intensity. To normalize the image, we used the most concentrated sample of an individual from the control as the standard, thus achieving a relative intensity.

### Enzyme-Linked Immunosorbent Assay

The DPP4/CD26 and the MMP9 concentrations were measured with the Human DPP4/CD26 DuoSet (DY1180) and Human MMP9 DuoSet (DY911-05) (R&D Systems, USA), respectively, according to the manufacturer’s instructions, both along with DuoSet ELISA Ancillary Reagent Kit 2.

### Sample Preparation for LC-MS/MS

#### In-Gel Protein Digestion

Among the 40 individual samples, 500 ng SWS proteins from three control, three pSS, and three sSS were loaded on 8% SDS-PAGE (**Supplementary Figure 1**). Gel slices were excised from each lane, reduced with 10 mM dithiothreitol (DTT) in 25 mM of ammonium bicarbonate buffer for 1 h at 56°C and alkylated by the addition of 55 mM iodoacetamide (IAA) in the same buffer for 45 min in the dark at 25°C. Samples were then washed with ACN and subsequently 25 mM of ammonium bicarbonate, lyophilized and dehydrated for trypsin digestion (12.5 ng/µl in buffer) for 18 h at 37°C. Peptides were extracted using a gradient of 0.1% TFA (v/v), 0.1% TFA (v/v) in 50% ACN (v/v) and 0.1% TFA (v/v) in 80% ACN (v/v). Samples were lyophilized and resuspended with 1% TFA (v/v), desalted on a pipette tip packed with a C18 membrane (Empore, Supelco) for mass spectrometry analysis. The in-gel protein digestion samples were resuspended in 0.1% (v/v) formic acid to proceed with the LC-MS/MS analysis.

#### In-Solution Protein Digestion

Samples were processed according to Poulsen (42) with some modifications. Briefly, salivary proteins of the three control, three pSS, and three sSS samples were resuspended in 6 M guanidine hydrochloride (Gnd-HCl) in 25 mM ammonium bicarbonate (pH 8.4). Samples were warmed at 70 °C for 5 min under agitation (1,000 RPM) using a Thermomixer (Eppendorf, Hamburg, Germany). Then, homogenates were centrifuged at 18,000 × g for 20 min, the debris discarded, and supernatants were collected for proteomics analysis. Next, the sample was quantified with Qubit^®^ 2.0 protein assay kit (Thermo Fisher Scientific, Maryland, USA). Aliquots of 150 µg (per condition/replicate) of whole saliva proteins were reduced with 20 mM dithiothreitol (DTT) in 0.20 mM ammonium bicarbonate pH 8.5 for 1 h at 56°C. They were then alkylated with 50 mM iodoacetamide for 1 h in the dark at room temperature. Subsequently, samples were diluted in 20 mM ammonium bicarbonate pH8.0 to a final concentration of Gnd-HCl of 0.9 M. Enzymatic digestion was performed with modified trypsin (Promega, Madison, WI, USA) at a ratio of 1:50 (enzyme: substrate) at 37°C overnight, followed by acidification with 0.1% (v/v) TFA. After the proteolysis, peptide samples were submitted to desalting on C18-reverse phase micro-columns, using self-prepared StageTips and vacuum-dried (43). After that, peptides were resuspended in 0.1% formic acid in the water and quantified by Qubit^®^ 2.0.

### LC-MS/MS

For both in-gel and in-solution protein digestion samples, the experiments were performed on a Dionex Ultimate™ 3000 RSLCnano system coupled online with an LTQ-Orbitrap Elite™ mass spectrometer (Thermo Scientific; San Jose, USA). Each peptide sample was loaded by an autosampler into the trap column at a flow rate of 4 μl.min^−1^ in 98% buffer A (0.1% formic acid in water) and 3.6% buffer B (0.1% formic acid in acetonitrile 80%). The peptides were separated at a constant flow 230 nl/min in a 20 cm analytical column (75 um inner diameter) packed with 3 µm C18 beads (Reprosil Pur-AQ, Dr. Maisch, Germany) with a 50 min gradient ranging for in-gel protein digestion samples and a 190 min gradient ranging for in-solution protein digestion samples from 5 to 35% acetonitrile in 0.1% formic acid, directly into mass spectrometer under ESI ionization.

Molecular mass spectra were acquired using Xcalibur 2.0 software (Thermo Fisher Scientific Inc., Waltham, MA, USA). Acquisition by the mass spectrometer was performed in data-dependent acquisition (DDA) mode. The DDA cycle consisted of a full scan in FTMS comprising a 400–1,800 m/z range under a resolution of 120,000 full widths at half-maximum at m/z 400. The 15 most abundant ions with an intensity of at least 3,000 counts were selected and fragmented by high energy collision dissociation (HCD). The fragmentation was performed with a collision energy of 35%, automatic gain control (AGC) of 1 × 10^6^ and acquired in an orbitrap analyzer with a 2 m/z isolation width and AGC 1 × 10^4^. Dynamic exclusion was set to 90 s. Ions with a charge state of +1 or undetermined were excluded.

### Mass Spectrometry Data Analysis

For both gel-based LC-MS/MS and gel-free LC-MS/MS, the raw files of each individual groups were searched with PEAKS Studio 7.0 (Bioinformatics Solutions Inc., Ontario, Canada) against the *Homo sapiens* database with 74,807 sequences downloaded from Uniprot on 01-27-2020. Retrieval parameter settings were as follows: Parent mass error tolerance 10 ppm; fragment mass error tolerance 0.5 Da; precursor mass search set as monoisotopic; enzyme as trypsin, number of proteins missed cleavages was set as two; cysteine alkylation was set as a fixed modification, variable modification as methionine oxidation. All the reported data were based on the 99% confidence interval for protein identification as determined by the false discovery rate (FDR) of 1% and at least one unique peptide for protein. The mass spectrometry and related data have been deposited to the ProteomeXchange Consortium (http://proteomecentral.proteomexchange.org) *via* the PRIDE partner repository (44) with the dataset identifiers PXD025434 and PXD025463, for gel-free and gel-based proteomic approaches, respectively. SignalP v.5.0 Server (http://www.cbs.dtu.dk/services/SignalP/) was used to predict proteins secreted by classical pathway. The parameter ‘eukaryotes’ was set to predict the secretion pathways. The Uniprot web server was required for conversions of gene list (45). Protein−protein interaction (PPI) was established by STRING (46) using UniProt Accession codes. The generated interaction networks were uploaded in Cytoscape 3.8.1 for graphical representation (47). Enrichment analysis was performed where gene ontology was over-represented. The Ensembl gene ID was used to feed g:Profiler (48).

### Statistical Analysis

GraphPad Prism for Mac (version 7.0e.198) or Statistical Package for Social Sciences (SPSS) for Windows (version 13.0) was used for all analyses, considering a p-value <0.05 as significant. The normal distribution of continuous variables was determined using D’Agostino & Pearson omnibus, Shapiro–Wilk, and Kolmogorov–Smirnov tests. For comparisons of numerical data between two groups, either Student’s t-test (*e.g*., age, protein concentration and immune enzymatic assays for Human DPP4/CD26) or Mann–Whitney test (*e.g.*, enzymatic activities and its intensity, and immune enzymatic assays for Human MMP9) were performed. Chi-square test was applied to compare the frequencies of categorical variables (*e.g.*, gender) in groups. Additionally, to compare protein concentration between groups, Student’s t-test and Levene test were used. We identified trends in the condition’s clinical stages and on immune enzymatic assays through Spearman’s correlation coefficient. Finally, a one-way ANOVA test and Bartlett’s test were performed to compare the dosage of DPP4 in subgroups, and a Kruskal–Wallis test to compare the dosage of MMP-9 in subgroups.

## Results

### Proteolytic Profile and Active DPP4/CD26 Detection in Saliva of SS Individuals

To adjust salivary protein concentration for mass spectrometry and evaluate proteolytic activity in standardized concentration, total protein concentration (ng/µl) was measured in both groups. Saliva from SS individuals had lower protein concentrations when compared to the control group (**Supplementary Figure 2**).

**Figure 1:**
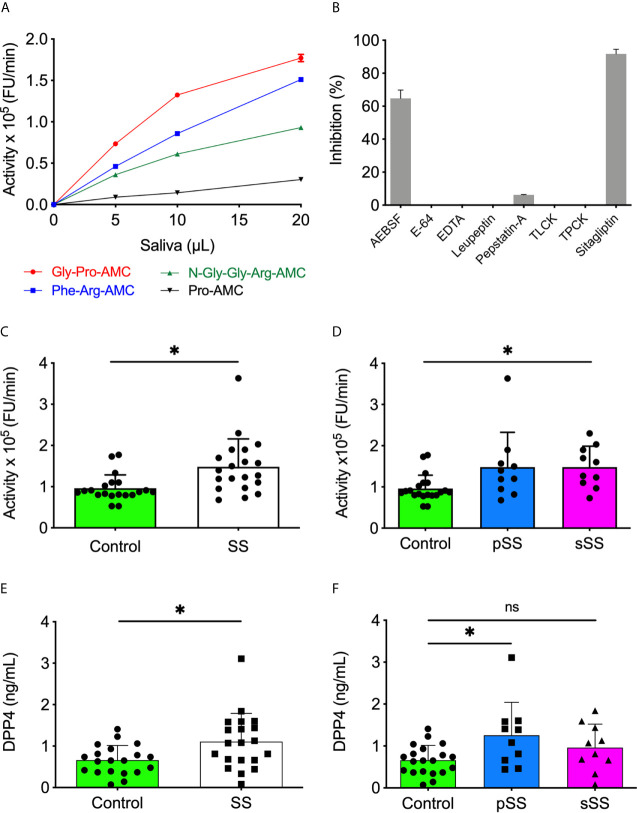
Increased levels and activity of DPP4/CD26 in saliva of Sjögren’s syndrome individuals. **(A)** The SWS samples (10 µl) were incubated with each substrate (20 µM) in HEPES 25 mM (pH 7.5). **(B)** The SWS samples were incubated with Gly-Pro-AMC (20 µM) in HEPES 25 mM (pH 7.5) in the presence of classical protease inhibitors or sitagliptin. **(C)** Proteolytic activity of SWS individuals and **(D)** between SS groups using Gly-Pro-AMC (20 µM) in HEPES 25 mM (pH 7.5). **(E)** The DPP4/CD26 concentration was measured in all saliva samples and **(F)** between SS groups with the Human DPP4/CD26 DuoSet ELISA kit (R&D Systems, USA). All experiments were performed in triplicate. Data are expressed as the mean ± standard deviation. *p < 0.05; Student’s *t*-test. **(D, F)** One-way A-NOVA and Dunnett’s multiple comparison test. ns, not significant.

Twelve fluorogenic substrates were tested on saliva from a control group individual for preliminary classification to characterize SS saliva’s proteolytic activity. Among them, Gly-Pro-AMC, Phe-Arg-AMC, N-Gly-Gly-Arg-AMC, and Pro-AMC had increased hydrolysis by proteases present in saliva (**Figure 1A**). The most expressive activity was observed with Gly-Pro-AMC, a highly specific substrate of DPP4. Considering the low volume of saliva samples collected due to SS patients’ hyposalivation, the low detection activity of Pro-AMC, and that several proteases can cleave Phe-Arg-AMC and N-Gly-Gly-Arg-AMC, only Gly-Pro-AMC was chosen to proceed within this study. Different classical protease inhibitors or sitagliptin, a highly selective inhibitor of DPP4, were incubated with SWS before proceeding to hydrolysis of Gly-Pro-AMC to confirm the specificity of DPP4 enzymatic activity in our assays. An expressive SWS proteolytic inhibition toward Gly-Pro-AMC was observed in the presence of AEBSF, a specific and irreversible serine protease inhibitor, while the other classical inhibitors did not affect the activity on Gly-Pro-AMC. However, sitagliptin completely abolished this activity in SWS (**Figure 1B**).

Subsequently, all forty SWS samples were tested using the DPP4 fluorogenic substrate. A significant (p < 0.05) increased proteolytic activity on Gly-Pro-AMC was observed in SS saliva, either pSS or sSS, in comparison to the control ones (**Figures 1C, D**). Besides detecting the DPP4 activity, an ELISA was performed to quantify soluble DPP4 in SWS samples. With a threshold detection of 0.02 ng/ml, an overexpression of DPP4 was reported in SS saliva (p < 0.05) (**Figure 1E**), specifically in pSS (**Figure 1F**). This result corroborates enzymatic assays and its inhibition shown in **Figures 1B, D**.

### Gelatinase Activity

A preliminary gelatin zymogram was performed to define the saliva volume to be used in this study. Ten microliter of saliva showed well defined gelatinolytic bands and was chosen to perform zymography in all forty saliva samples (**Supplementary Figure 3**). Mirrored zymography to a 10% SDS-PAGE silver-stained loaded with 500 ng was performed. Hence, proteolytic activity was measured over total protein concentration. We found the same ratio of proteolytic activity intensity when loading the gel by volume of saliva (**Supplementary Figure 4**).

**Figure 2:**
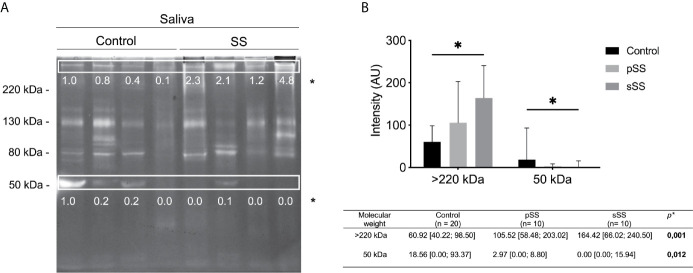
Gelatinolytic activity profile in saliva from healthy and Sjögren’s syndrome individuals. **(A)** Protease activity analysis of 10 μl SWS proteins (per lane) from healthy or SS individuals. Gels (8% polyacrylamide with 0.1% gelatin, w/v) were stained with Coomassie Brilliant Blue R250. Clear bands on dark background indicate sites of protein degradation. Spearman’s correlation test. Molecular weight markers (kDa). **(B)** The intensity of activity bands was assessed by densitometry using the software ImageJ. The most concentrated sample from a control individual was used as the standard to achieve the relative intensity to normalize the image. Mann–Whitney test. *p < 0.05.

We analyzed all forty saliva samples on zymography, following the same distribution exemplified in **Figure 2A**. Thereby, SWS samples revealed a distinct pattern between groups, both in molecular weight (kDa) and gelatinolytic activity intensities. The molecular weight and intensity of bands were then cataloged and evaluated. We initially identified 10 bands and analyzed their frequency statistically. No differences were found between groups (p > 0.05), according to the Mann–Whitney test (**Supplementary Figure 5**).

Regarding the intensity of bands, there was a significant difference (p < 0.05) in the intensity of gelatinolytic activity of >220 and 50 kDa bands, according to the Mann–Whitney test (**Figure 2A**). The >220 kDa band exhibits an increase in intensity among SS individuals. Meanwhile, the 50 kDa band shows a reduction of intensity among SS individuals. Thereby, these proteolytic bands presented significant differences regarding clinical condition, pSS and sSS (**Figure 2B**). Although we used individuals’ age for correlation analyses, this variable was not relevant (no significant results) for these tests. Gender could not be analyzed, as there was only one male per group.

**Figure 3:**
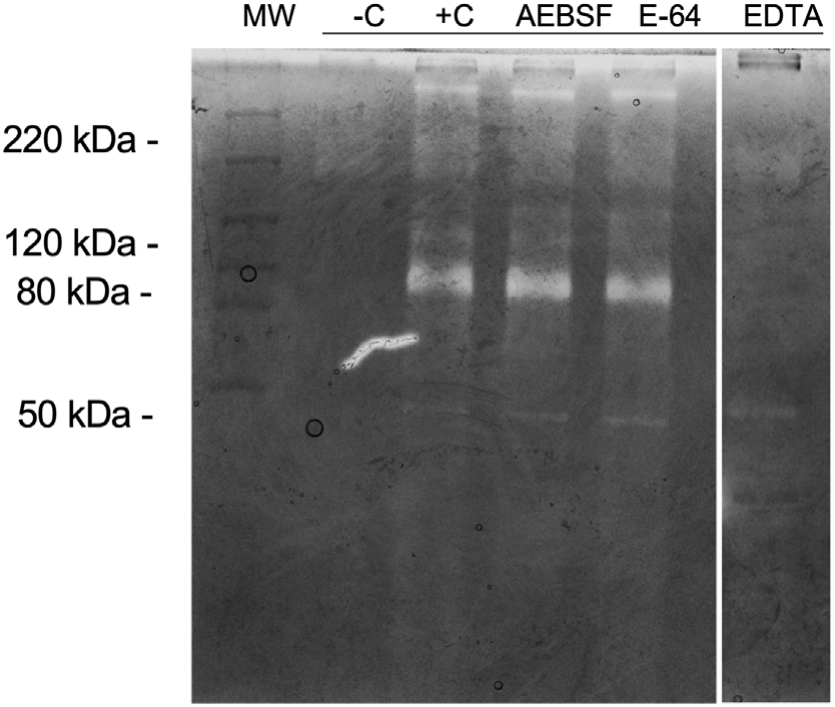
Inhibition of proteolytic activity in gelatin zymography. Protease activity analysis of 10 μl SWS proteins (per lane). Gel strips (8% polyacrylamide with 0.1% gelatin, w/v) were incubated in the presence of AEBSF (1 mM), E-64 (100 µM) or EDTA (1 mM). Strips were stained with Coomassie Brilliant Blue R250. Clear bands on dark background indicate sites of protein degradation. -C: Boiled SWS. Molecular weight markers (kDa).

We analyzed proteolytic inhibition using the following specific inhibitors: AEBSF for serine proteases; E-64 for cysteine proteases; and EDTA, for metalloproteases, to determine the protease class involved in zymography bands from SWS. Our results show that AEBSF and E-64 could not inhibit gelatinolytic activities since the proteolytic profile exhibits similarity with the positive control (+C). However, EDTA inhibited most proteolytic bands, except the 40 kDa band (**Figure 3**).

**Figure 4:**
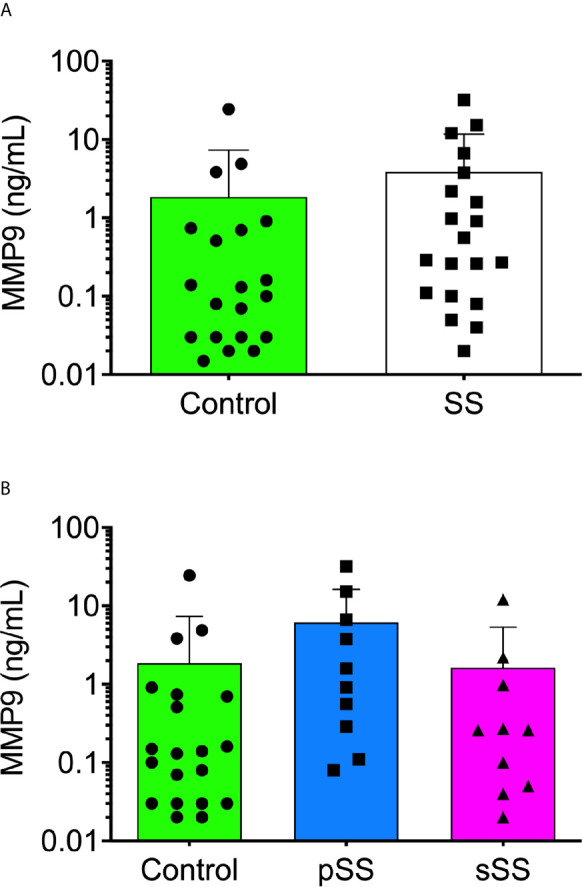
Detection of human MMP9 in the saliva from Sjögren’s syndrome individuals. The MMP9 concentration was measured in all saliva samples [**(A)**; Mann–Whitney test] and between SS groups [**(B)**; Kruskal–Wallis test] with the Human MMP9 DuoSet ELISA kit (R&D Systems, USA) according to the manufacturer’s instructions.

Among the metalloproteases with gelatinolytic activity, MMP9 has been implicated in molecular mechanisms of several auto-immune syndromes (49). To assess the MMP9 expression profile in SWS, an ELISA was performed. With a threshold detection of 0.02 ng/ml, the ELISA result reported a tendency for significance (p = 0.0531), according to the Mann–Whitney test (**Figure 4A**), and there was a tendency for significance (p = 0.0527) in control with pSS samples (**Figure 4B**), according to the Kruskal–Wallis test. This evidence corroborated enzymatic assays and their inhibition since MMP9 is a gelatinase, and there was higher expression (p < 0.05) in SS samples for >220 kDa proteolytic band.

### Correlation Between DPP4 and MMP9

**Figure 5:**
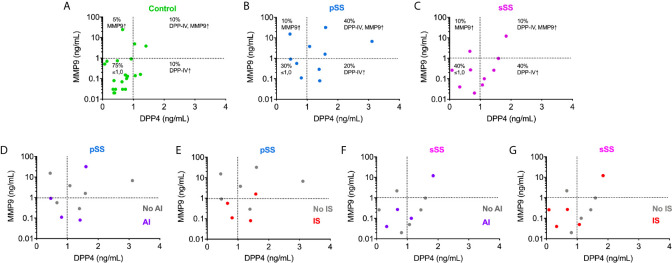
Immunoassay measurements for MMP9 and DPP4/CD26 illustrate the interdependence between these proteases in the saliva from pSS individuals. Analysis of correlation of MMP9 concentrations with those of DPP4/CD26 in control **(A)**; pSS: primary Sjögren’s syndrome **(B, D, E)**; and sSS: secondary Sjögren’s syndrome **(C, F, G)** individuals. AI, anti-inflammatory drugs use; No AI, no anti-inflammatory drugs use; IS, immunosuppressive drugs use; No IS, no immunosuppressive drugs use.

Although we did not observe an essential interdependence between MMP9 and DPP4 concentrations for SS patients in the global analysis. When the test cut-off value was set at >1 ng/ml, the results demonstrated that MMP9 and DPP4 directly correlated in approximately 40% of SWS samples from pSS patients, who presented higher levels of both proteases (**Figure 5**). SS patients were grouped according to the drugs they were taking to control the disease (**Supplementary Table 3**). Anti-inflammatory and immunosuppressive drugs showed no influence on the concentrations of MMP9 and DPP4/CD26 (**Figure 5**). We found no significant data in either DPP4 or MMP9 average values when compared with the medicament used.

### Proteases and Protease Inhibitors Identified in SS Saliva

**Figure 6:**
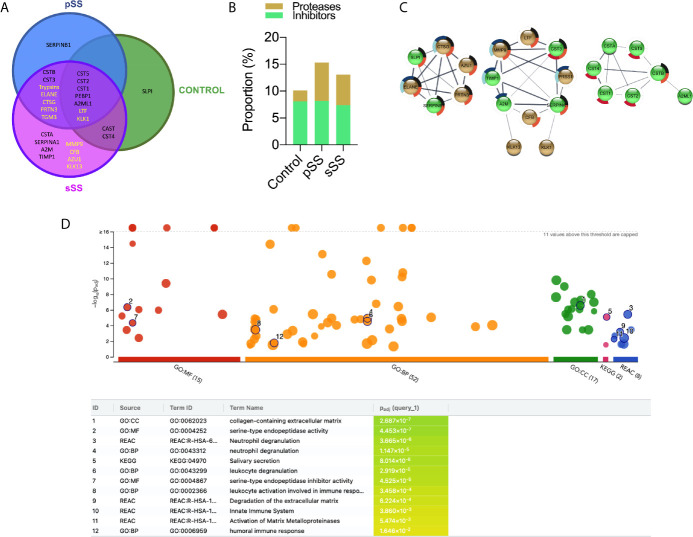
Proteases and protease inhibitors identified in Sjögren’s syndrome saliva by LC-MS/MS. **(A)** Venn diagram for the proteases (yellow font) and protease inhibitors (black font) identified commonly or exclusively among the three groups. **(B)** Proportion of proteases and protease inhibitors identified in each comparison group. **(C)** Protein–protein interaction (PPI) analysis in STRING database. A confidence score of 0.4 was set as a cut-off allowing active interaction sources as evidence. Line thickness indicates the strength of data support. Brown: proteases. Green: protease inhibitors. Pie chart colors are related to the enrichment analysis performance by g:Profiler. Black: neutrophil degranulation (GO.0043312); dark blue: degradation of the extracellular matrix (REAC:R-HSA-1474228); light blue: activation of matrix metalloproteinases (REAC:R-HSA-1592389); red: salivary secretion (KEGG:04970) and orange: immune response (GO:0006955). **(D)** g:Profiler enrichment analysis plot. GO : MF (Molecular Function), GO : BP (Biological Process), GO : CC (Cellular Component), and KEGG Pathways. The number in the source name in the x-axis labels shows how many significantly enriched terms were found. Black circled numbers inside the plot are related to the ID column in the table.

To examine the overall panorama of proteases in saliva, samples from pSS and sSS patients *versus* the control individuals were analyzed by LC-MS/MS. Two different approaches, gel-based LC-MS/MS and gel-free LC-MS/MS that comprise in-gel digestion and in-solution digestion, respectively, were employed to increase the amount of identified proteins. Combined results of both approaches for each individual group resulted in 99 protein groups (PGs) in control group, 98 in pSS and 176 in sSS. Regarding proteases and protease inhibitors, 10 PGs were identified in the control group, 15 in pSS and 23 in sSS. The proportion of protease inhibitors found among the three groups did not differ (Control: 8.1, pSS: 8.2, and sSS: 7.4). However, an increase in protease identifications was noticed in SS groups, mainly in pSS (Control: 2.0, pSS: 7.1, and sSS: 5.7; **Figure 6**). Secreted proteins can be predicted by the presence of a N-terminal cleavable signal peptide that is typically 15–30 amino acids long. Herein, *in silico* prediction of secretion of proteases and protease inhibitors through the classical pathway showed that 100% of these PGs were predicted to be secreted (**Supplementary Table 4**).

Protease PGs neutrophil elastase (ELANE), cathepsin G (CTSG), trypsin (PRSSs), and myeloblastin (PRTN3), which are all serine proteases, were identified only in SS samples. Likewise, protein-glutamine gamma-glutamyltransferase E (TGM3) that contains a cysteine protease domain from the C111.003 family (MEROPS), was found only in both SS groups. Remarkably, MMP9, complement factor B (CFB), azurocidin (AZU1) and kallikrein-13 (KLK13) were detected exclusively in sSS. No exclusive protease was identified in control or pSS samples (**Figure 6A**). Nevertheless, DPP4/CD26 was not reported among the three groups by proteomic analysis.

Additionally, we found several cysteine proteases inhibitors of cystatins family (CST1, CST2, CST3, CST4, CST5, CSTA, CSTB, CTSG), serine/cysteine protease serpins (SERPINB1 and SERPINA1), the broad-spectrum endopeptidase-binding alpha-2 macroglobulin (A2M), the serine protease phosphatidylethanolamine-binding protein (PEBP1), calpain inhibitor calpastatin unit 1 (CAST) and the tissue inhibitor of metallopeptidases-1 (TIMP-1).

PPI network was generated using STRING database followed by MCL cluster in order to associate protein of related functions (**Figure 6C**). The left network clustered the serine proteases ELANE, CTSG, and PRTN3 that seem to be involved with innate immune response (50). The middle network clustered proteases related to ECM remodeling (MMP9 and KLK13), inflammation (MMP9 and PRSS1 (Trypsin-1)) and complement pathway (CFB). Interestingly, these proteases were identified exclusively in SS, reinforcing their involvement in SS pathogenesis. Finally, the right network grouped the cysteine proteases inhibitors of cystatins family, which are known to be present in human saliva (51). We also performed an enrichment analysis where gene ontology is over-represented. The Ensembl gene IDs were used to feed g:Profiler (48), and the most expressive results are listed in **Figure 6D**, with their respective p values. Pie chart colors inside **Figure 6C** highlight which proteases and inhibitor proteases are classified into the enrichment analysis terms.

## Discussion

Sjögren’s syndrome is an autoimmune exocrinopathy that affects primarily women, with first symptoms appearing in ages ranging from the fourth to sixth decades of life (7). Hence, most of the present study participants were women (38 out of 40 individuals), which accurately represents the world population affected by SS (52).

Here we report for the first time that DPP4/CD26 is upregulated in SS patient saliva. DPP4 or T-cell activation antigen CD26 (EC 3.4.14.5) is an exopeptidase of the prolyl-oligopeptidase family, which belongs to the class of serine proteases (53) and cleaves proline or alanine amino acids from the N-terminal side of peptides (54). It is a widely distributed multifunctional integral membrane protein, but can also be cleaved releasing its soluble extracellular domain in body fluids (*e.g.* plasma and serum) (55). DPP4/CD26’s most notable function is in glucose homeostasis through regulation of the incretin hormones (56). Besides its proteolytic role, DPP4/CD26 acts as a cell surface receptor, signal transduction mediator, adhesion and costimulatory protein (57). Regarding the former, DPP4/CD26 contributes to T lymphocyte activation and antigen-presenting cell–T-cell interaction (58–60), having a significant role in many autoimmune and inflammatory diseases (37). The overexpressed DPP4/CD26 in pSS saliva may play a role in the development of the disease since cytokines and chemokines are the main substrates for the enzyme (61). Post-translational modification of those molecules by proteases is an essential regulatory tool to enhance or dampen the inflammatory response. In human mucosal-associated invariant T (MAIT) cells, DPP4/CD26 expression levels are high (62). These cells are expanded in the salivary glands of SS patients and may be deleterious *via* IL-17 production (63), which has pathogenic roles in many autoimmune diseases (64). DPP4/CD26 knockout in mice with lung transplantation resulted in a significant reduction of IL-17 and IL-21, Th17 cytokines (65). Thus, it is feasible that in MAIT cells from SS patients, IL-17 might be processed by DPP4/CD26. On the other hand, saliva from sSS patients present the same level of DPP4/CD26 activity when compared to healthy controls. DPP4/CD26 levels are decreased in serum of patients diagnosed with the commonly systemic autoimmune diseases, such as rheumatoid arthritis and systemic lupus erythematosus, which occur along with sSS (66). Worth of note, most of the analyses regarding DPP4/CD26 activity available in the literature were carried out based on experiments using patient serum. In this sense, our investigation reinforces saliva as an attractive biofluid and an alternative to serum/blood as a supply of material for the prognosis, diagnosis and treatment of oral diseases (67).

Although mass spectrometry analysis could not identify DPP4 in any SWS, ELISA and proteolytic assays testify its upregulation in SS samples. Thus, a possible explanation for the absence of detection of DPP4/CD26 could be the lower concentration of endogenous proteases compared to constitutive proteins (68). Since DPP4/CD26 is an integral membrane protein, it could be lost during sample processing for LC-MS/MS analysis. Moreover, DPP4/CD26 is considerably glycosylated, what turns it difficult to digest by trypsin for mass spectrometry analysis (69, 70).

Among serine proteases revealed by mass spectrometry, neutrophil elastase (ELANE), cathepsin G (CTSG), and myeloblastin (PRTN3) were found only in SS saliva samples compared to control ones. These proteases belong to the chymotrypsin superfamily and are enriched within the azurophilic granules from polymorphonuclear neutrophils (PMN). When activated, PMN releases the neutrophil extracellular traps (NETs) containing these protease as long as myeloperoxidase, also found in MS/LS results for SS individuals (71). These complexes have been reported in autoimmune diseases, such as rheumatoid arthritis (72). ELANE is one of the most damaging enzymes in the body and a great release can cause local tissue injury (73). CTSG activates metalloproteases and cleaves extracellular matrix proteins, contributing to neutrophil migration (74), stimulates the production of cytokines and chemokines (75, 76), and seems to be important in regulating the balance between tissue protection and damage during inflammation (77). PRTN3 is involved in granulocyte differentiation (78), and once augmented, it can negatively affect the resolution of inflammation that causes immune system deregulation (79). It was also shown to be involved in cell death induction after caspase 3 activation in a mouse model and cell culture (80). These three serine proteases increased expressions were also reported in chronic obstructive pulmonary disease (COPD) patients (81), which is common in pSS patients, even in those who have never been smokers (82).

According to MEROPS, peptide sequence Gly-Pro is not a substrate hydrolyzed by ELANE, CTSG or PRTN3. Nevertheless, ELANE is related to the digestion of extracellular matrix components by PMN, such as Pro-Gly-Phe-Gly-Gly-Pro-Asn-Cys (laminin subunit gamma-2) and Leu-Gly-Pro-Val-Thr-Pro-Glu-Ile (matrix metalloproteinase-2), which plays a role in inflammation and remodeling tissue by secretion of pro-inflammatory factors (83). Besides, it has been suggested ELANE might be part of the posttranslational processing of an MMP2 (84). Also, elastase produced by defense cells could perform proteolytic destruction of cartilage in rheumatoid arthritis, a disease prevalent in sSS (85).

Gelatinases degrade extracellular matrix components such as collagen, fibronectin and laminin and nonmatrix substrates, such as serpins, tissue factor pathway inhibitor and insulin-like growth factor binding proteins (86). We noticed that most of the activity bands in gelatin zymograms were inhibited by the metalloprotease inhibitor EDTA, from approximately 90 kDa bands whose molecular weight were similar to the active form 86 kDa MMP9 (87). The MMP9 was identified by LC-MS/MS only in sSS saliva. MMPs are expressed in many circumstances and when there is an imbalance between the expression of these enzymes and their inhibitors, there may be a pathological process in which inflammatory response and tissue remodeling, and migration cell growth are observed. Besides MMP9, KLK13 may also cleave extracellular matrix proteins, influencing tissue remodeling (88). This functional association is reflected in the PPI network since these proteases were grouped in the same cluster (**Figure 6C**; middle cluster). During the inflammatory process of SS, MMP1, MMP2, MMP3, and MMP9 are released (89). Also, high MMP9 expression and activity have been detected in the saliva, tear and the labial salivary glands of SS patients (47, 90, 91). The former was correlated with structural and functional glandular impairment in severe, active pSS patients (92). In the context of a previous report in pSS (93), high plasma MMP9 was indicative of definite pSS, although in the same study MMP9 polymorphism could not be used for pSS risk assessment. Additionally, metalloproteinase inhibitors have been found endogenously in SS saliva samples, such as TIMP-1 thus leaving it clear that the activity of MMP9 had been modulated (94).

A positive correlation was noticed between DPP4/CD26 and MMP9 dosage in 40% pSS saliva samples, a connection that has already been reported in prostate cancer. In that case, results indicated DPP4 initiates signal transduction and consequently regulates the MMP9 expression (95). Also, since no correlation between the use of medicines and the proteases dosage was reported, it may be useful information for future analysis as biomarkers.

The Analysis in g:Profiler revealed some relevant results. The neutrophil degranulation in innate immune response reaffirms the possibility of NETs involvement. The mass spectrometry analysis reported MMP9 and serine proteases involved in innate immune response in SS samples only (ELANE, CTSG, and PRTN3) (50). A strong association between these three enzymes was also evidenced in our PPI network (**Figure 6C**; left cluster). Additionally, leukocyte degranulation may refer to lymphocytic infiltration in SS individuals’ glandular tissue (96).

The data presented in this study highlight the usefulness of proteases in the saliva from SS patients as biomarkers. We propose here that DPP4/CD26 activity and concentration in human saliva could be explored as a potential salivary biomarker in diagnosing SS, mainly pSS. Moreover, through proteomic analysis, ELANE, CTSG, and PRTN3 measurements, which were found only in the saliva from SS patients, could significantly improve the ability to distinguish SS patients from healthy subjects. New insights into these protease biological roles in the pathogenesis of SS are necessary to design therapeutic approaches based on protease inhibition.

## Data Availability Statement

The datasets presented in this study can be found in online repositories. The names of the repository/repositories and accession number(s) can be found below: https://www.ebi.ac.uk/pride/archive/, PXD025434 https://www.ebi.ac.uk/pride/archive/, PXD025463.

## Ethics Statement

The studies involving human participants were reviewed and approved by the Ethics Committee of the University of Brasilia, CEP/FS, 073/11. The patients/participants provided their written informed consent to participate in this study.

## Author Contributions

LG, SM, AT, and AA performed the experiments. IB, AT, and SC designed the study and supervised the experiments. LG, IB, FM, CA, SC, and ON interpreted the data. MS, SC, and JS performed the LC-MS/MS analysis. FMBM and LP collected and prepared saliva samples. LG, FM, CA, IB, and SC prepared the manuscript. All authors contributed to the article and approved the submitted version.

## Funding

This work was supported by grants and fellowships awarded by the Fundação de Amparo à Pesquisa do Distrito Federal (FAP- DF, 0193.001803/2017 and 0193.001485/2017), Coordenação de Aperfeiçoamento de Pessoal de Nıv́el Superior (CAPES, grant 923/18 CAPES-COFECUB), Conselho Nacional de Desenvolvimento Científico e Tecnológico (CNPq, INCT- MCTI/CNPq/FAPs 16/2014). LG and SM received scholarships from CNPq and CAPES. The funders had no role in study design, data collection and interpretation, or the decision to submit the work for publication.

## Conflict of Interest

The authors declare that the research was conducted in the absence of any commercial or financial relationships that could be construed as a potential conflict of interest.
