# Supplementary material for: Mapping Salivary Proteases in Sjögren’s Syndrome Patients Reveals Overexpression of Dipeptidyl Peptidase-4/CD26
Source: Front Immunol. 2021 Jun 17;12:686480. doi: 10.3389/fimmu.2021.686480 (PMC8247581; doi:10.3389/fimmu.2021.686480)
Supplement: Supplementary file 1 [file DataSheet_1.pdf]

# Mapping Salivary Proteases in Sjögren's Syndrome Patients Reveals Overexpression of Dipeptidyl Peptidase-4/CD26

**Laís Garreto, Sébastien Charneau, Samuel Mandacaru, Otávio T. Nóbrega, Flávia N. Motta, Carla N. de Araújo, Audrey C. Tonet, Flávia M. B. Modesto, Lilian M. de Paula, Marcelo Valle de Sousa, Jaime M. Santana, Ana Carolina Acevedo, Izabela M. D. Bastos***

*** Correspondence:**
Izabela M. D. Bastos
dourado@unb.br

## Supplementary Figure Captions

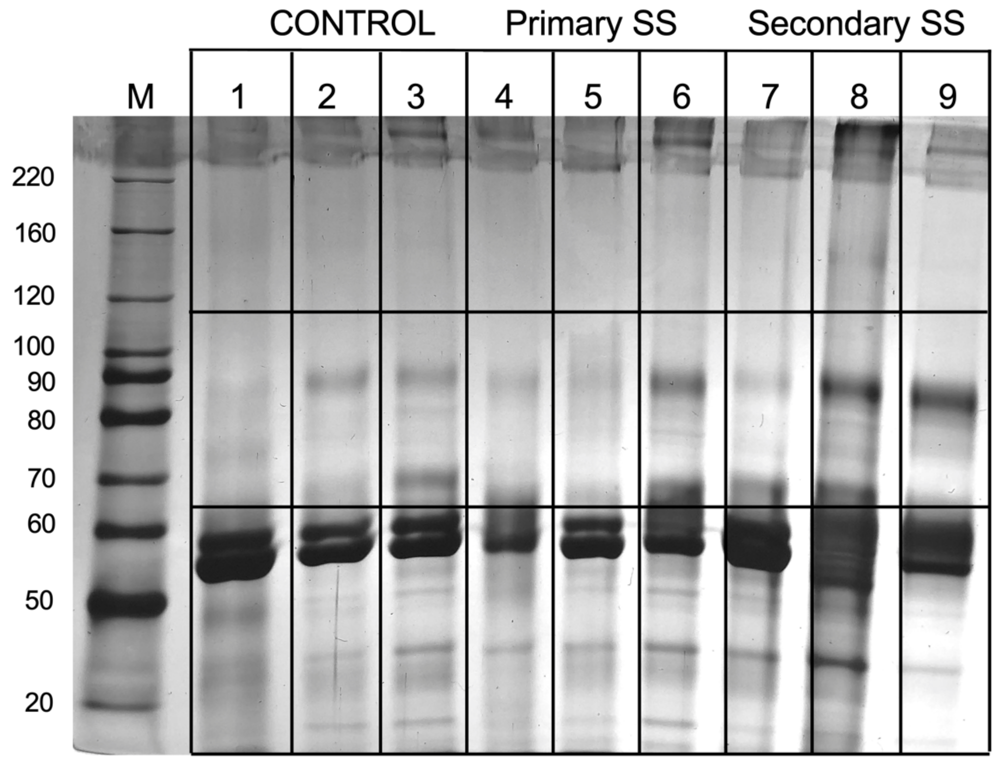


**Supplementary Figure 1. Protein bands in saliva from healthy or Sjögren's syndrome individuals.** SDS-PAGE 8% followed by silver staining (500 ng of SWS per lane). Protein bands were excised from gel for LC-MS/MS analysis. The samples were not boiled prior to gel loading.

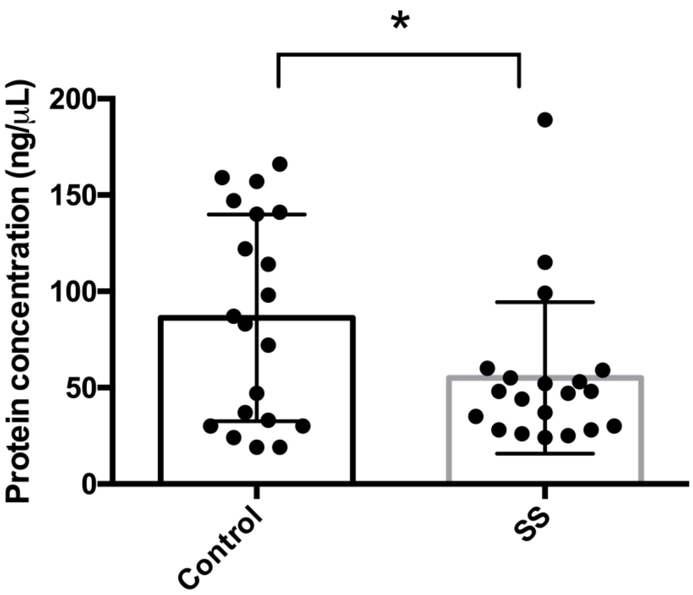


**Supplementary Figure 2. Total proteins in saliva from healthy or Sjögren's syndrome individuals.** Total salivary protein concentration was determined by fluorometric assay (Invitrogen Qubit® 2.0 Fluorometer) with the Invitrogen™ Qubit™ Protein Assay Kit. Student's t- and Levene tests. *Significance threshold set at p<0.05.

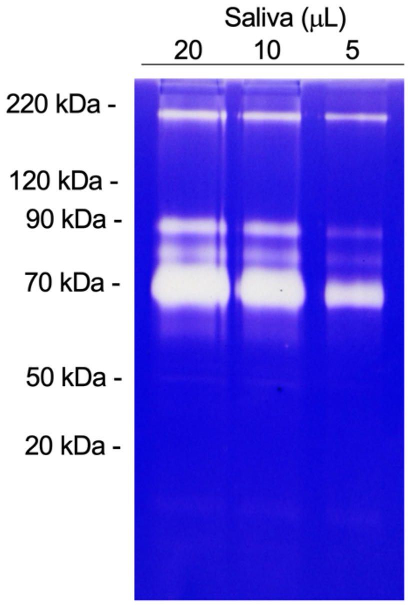


**Supplementary Figure 3. Gelatinolytic activity profile in saliva from a healthy individual.** Protease activity analysis of 20, 10 and 5 μL SWS proteins (per lane) from a healthy individual. Gel (8 % prolyacrylamide with 0.1 % gelatin, w/v) was stained with Coomassie Brilliant Blue R250. Clear bands on blue background indicate sites of protein degradation. Molecular weight markers (kDa).

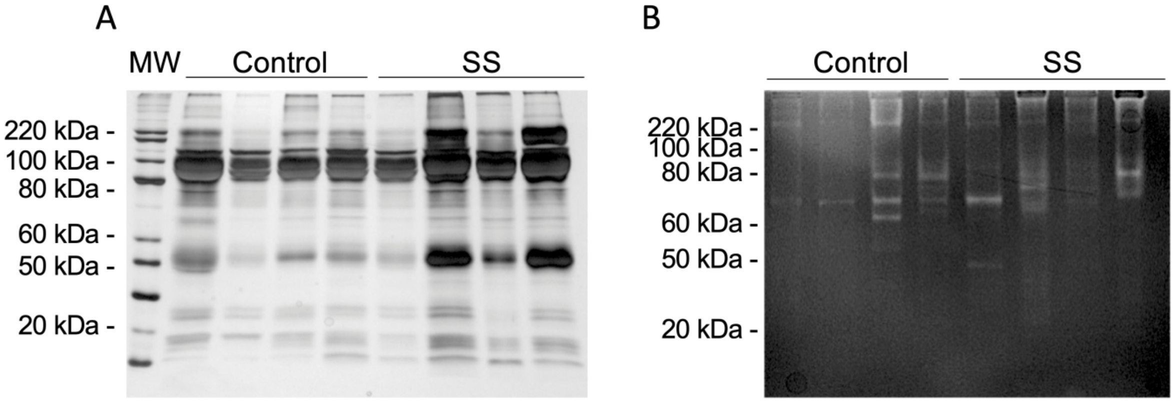


**Supplementary Figure 4. Protein and gelatinolytic activity profiles in saliva from healthy or Sjögren's syndrome individuals.** (A) SDS-PAGE 10% followed by silver staining (500 ng of SWS per lane). The samples were not boiled prior to gel loading in order to reveal similar protein profile to zymograms. (B) Protease activity analysis of 500 ng SWS proteins (per lane) from healthy or SS individuals. Gels (8 % prolyacrylamide with 0.1 % gelatin, w/v) were stained with Coomassie Brilliant Blue R250. Molecular weight markers (kDa). Both gels were run simultaneously, with the same SWS samples from healthy and SS individuals.

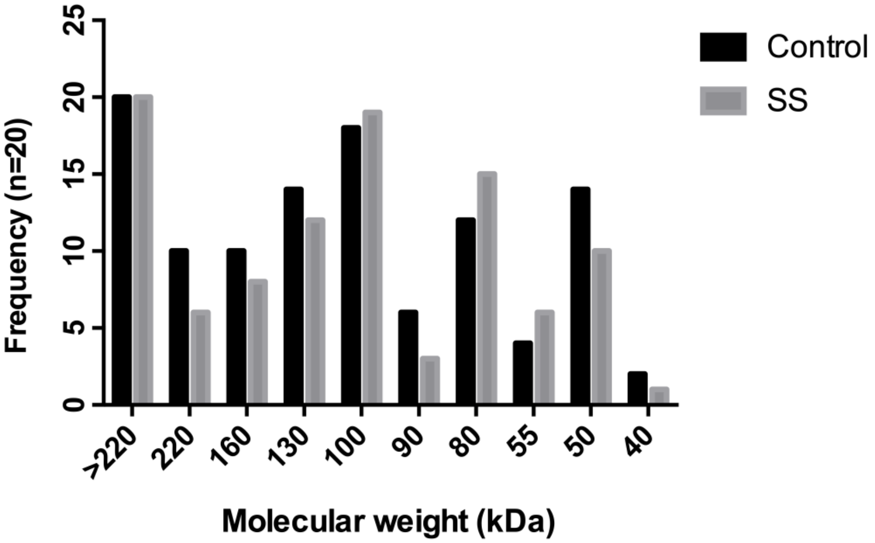


**Supplementary Figure 5. Comparison of the molecular weight of gelatinolytic bands in the saliva from healthy individuals and patients with Sjögren's syndrome.** No statistically significant difference between groups, Mann-Whitney test ($p>0.05$).
