# Supplementary material for: Mapping Salivary Proteases in Sjögren’s Syndrome Patients Reveals Overexpression of Dipeptidyl Peptidase-4/CD26
Source: Front Immunol. 2021 Jun 17;12:686480. doi: 10.3389/fimmu.2021.686480 (PMC8247581; doi:10.3389/fimmu.2021.686480)
Supplement: Supplementary file 2 [file Table_1.docx]

| **Feature** | | |  | **Individuals** | | |
| --- | --- | --- | --- | --- | --- | --- |
|  |  |  |  | Control | pSS | sSS |
|  |  |  |  | (n=20) | (n=10) | (n=10) |
| **Age (years)** | | |  | 47.7 ± 8.84 | 55.4 ± 10.83 | 48.4 ± 9.75 |
| **Duration of disease (years)** | | | | 0 | 5.5 ± 2.84 | 7.8 ± 4.78 |
| **Time till the diagnosis (years)** | | | | 0 | 2.5 ± 2.37 | 4.8 ± 5.61 |
| **Appearance of oral mucosa** | | | |  |  |  |
| Normal | |  | 20 | 5 | 3 |  |
| Dry | |  | 0 | 5 | 7 |  |
| **Clinical appearance of tongue** | | | |  |  |  |
| Normal | |  | 17 | 5 | 3 |  |
| Dry | |  | 0 | 2 | 0 |  |
| Atrophic | |  | 0 | 1 | 2 |  |
| Atrophic with fissures | | | 0 | 1 | 1 |  |
| Savory | |  | 3 | 1 | 4 |  |
| **Saliva aspect** | | |  |  |  |  |
| Normal | |  | 19 | 2 | 1 |  |
| Sticky | |  | 1 | 3 | 1 |  |
| Foamy | |  | 0 | 5 | 8 |  |
| **Clinical presense of Candidiasis** | | | |  |  |  |
| No signs | |  | 15 | 8 | 5 |  |
| Angular cheilitis | | | 0 | 1 | 0 |  |
| Erytematous lesions | | | 5 | 1 | 5 |  |
| **Other autoimmune diseases** | | | |  |  |  |
| Lupus erythematosus | | | 0 | 0 | 3 |  |
| Rheumatoid arthritis | | | 0 | 0 | 7 |  |
|  | |  | |  |  |  |
|  | |  | |  |  |  |

Supplementary Table 1. Age and clinical information of participants.
