# Supplementary material for: Mapping Salivary Proteases in Sjögren’s Syndrome Patients Reveals Overexpression of Dipeptidyl Peptidase-4/CD26
Source: Front Immunol. 2021 Jun 17;12:686480. doi: 10.3389/fimmu.2021.686480 (PMC8247581; doi:10.3389/fimmu.2021.686480)
Supplement: Supplementary file 3 [file Table_2.docx]

**Supplementary Table 2. Protease inhibitors used in the fluorogenic and zymographic assays.**

| **Protease catalytic type** | **Inhibitor** | **Final concentration** |
| --- | --- | --- |
| Serine protease | AEBSF | 1 mM |
| Cysteine protease | E-64 | 100 μM |
| Metalloprotease | EDTA | 1 mM |
| Cysteine protease  Serine protease  Threonine protease | Leupeptin | 100 μM |
| Aspartic Acid | Pepstatin A | 100 μM |
| Dipeptidyl Peptidase IV | Sitagliptin | 1 μM |
| Cysteine protease  Serine protease | TLCK | 100 μM |
| Cysteine protease  Serine protease | TPCK | 100 μM |
