# Supplementary material for: Mapping Salivary Proteases in Sjögren’s Syndrome Patients Reveals Overexpression of Dipeptidyl Peptidase-4/CD26
Source: Front Immunol. 2021 Jun 17;12:686480. doi: 10.3389/fimmu.2021.686480 (PMC8247581; doi:10.3389/fimmu.2021.686480)
Supplement: Supplementary file 4 [file Table_3.docx]

**Supplementary Table 3. List of medications in use by the Sjögren’s syndrome patients.**

| **Drugs** | **pSS** | **sSS** |
| --- | --- | --- |
| Corticosteroids | 4 | 1 |
| Hydroxychloroquine | 1 | 4 |
| Immunosupressive | 4 | 1 |
| Pilocarpine | 1 | 0 |
| Eye drops | 8 | 6 |
| Anti-depressive | 6 | 3 |
| Anti-inflammatory | 4 | 1 |
| Other | 9 | 8 |
